# Benzanthric Acid, a Novel Metabolite From *Streptomyces albus* Del14 Expressing the Nybomycin Gene Cluster

**DOI:** 10.3389/fchem.2019.00896

**Published:** 2020-01-10

**Authors:** Marta Rodríguez Estévez, Nils Gummerlich, Maksym Myronovskyi, Josef Zapp, Andriy Luzhetskyy

**Affiliations:** ^1^Pharmaceutical Biotechnology, University of Saarland, Saarbrücken, Germany; ^2^Department of Pharmacy, Institute of Pharmaceutical Biology, University of Saarland, Saarbrücken, Germany; ^3^Helmholtz Institute for Pharmaceutical Research Saarland, Saarbrücken, Germany

**Keywords:** benzanthric acid, biosynthetic gene cluster, heterologous expression, nybomycin, secondary metabolite, *Streptomyces*

## Abstract

Streptomycetes constitute a diverse bacterial group able to produce a wide variety of secondary metabolites with potential applications in the pharmacy industry. However, the genes responsible for the biosynthesis of these compounds are very frequently inactive or expressed at very low levels under standard laboratory cultivation conditions. Therefore, the activation or upregulation of secondary metabolite biosynthesis genes is a crucial step for the discovery of new bioactive natural products. We have recently reported the discovery of the biosynthetic genes for the antibiotic nybomycin (*nyb* genes) in *Streptomyces albus* subsp. *chlorinus*. The *nyb* genes were expressed in the heterologous host *Streptomyces albus* Del14, which produces not only nybomycin, but also a novel compound. In this study, we describe the isolation, purification, and structure elucidation of the new substance named benzanthric acid.

## Introduction

Numerous species of the actinomycetal genus *Streptomyces* harbor metabolic pathways that produce secondary metabolites with a broad spectrum of bioactivities, which in many cases represent potential drug leads for the development of pharmaceuticals (Paulus et al., 2017; Protasov et al., 2017). The biosynthetic genes for these metabolites are generally arranged in clusters whose expression is often very low under laboratory cultivation conditions. Therefore, diverse strategies have been developed in order to induce the biosynthesis of natural products in *Streptomyces* strains (McKenzie et al., 2010; Olano et al., 2014; English et al., 2017). A widely used approach consists in the heterologous expression of a specific gene cluster in an optimized host strain where some of the native secondary metabolite genes have been removed (Myronovskyi et al., 2018; Bu et al., 2019). The simplified metabolic background promotes the channeling of biosynthetic precursors toward the production of the heterologous metabolites and facilitates their identification.

The marine strain *Streptomyces albus* subsp. *chlorinus* NRRL B-24108 is responsible for the production of bioactive metabolites such as the herbicide albucidin and the antibiotic nybomycin (Hahn et al., 2009; Rodriguez Estevez et al., 2018). We have recently discovered the biosynthetic genes for nybomycin (*nyb* genes) in *S. albus* subsp. *chlorinus* through a heterologous expression approach using the chassis strain *Streptomyces albus* Del14 as a host (Rodriguez Estevez et al., 2018). Besides nybomycin, we detected a new metabolite in the extract of *S. albus* 4N24 expressing the *nyb* genes. In this study, we report the isolation, purification and structure elucidation of the novel compound, benzanthric acid, from the culture of *Streptomyces albus* 4N24.

## Materials and Methods

### General Experimental Procedures

*Streptomyces* and *Escherichia coli* strains used in this work are listed in Supplementary Table 1. LB medium was used for cultivation of *E. coli* strains according to standard protocols (Green and Sambrook, 2012). *Streptomyces* strains were cultivated in standard media (Kieser et al., 2000): soy flour mannitol agar (MS agar) and liquid tryptic soy broth (TSB; Sigma-Aldrich, St. Louis, MO, USA). Additionally, liquid DNPM medium (40 g/L dextrin, 7.5 g/L soytone, 5 g/L baking yeast, and 21 g/L MOPS, pH 6.8) was used for secondary metabolite expression. The following antibiotics were supplemented when required at concentrations of 50 μg/μl (solid medium) or 25 μg/μl (liquid medium): kanamycin, apramycin, and nalidixic acid (Carl Roth, Germany; Sigma-Aldrich, USA).

### Isolation and Manipulation of DNA

Bacterial artificial chromosome (BAC) 4N24 was isolated from a library comprising the genome of *Streptomyces albus* subsp. *chlorinus* (Intact Genomics, St. Louis, MO, USA). DNA manipulations and cloning procedures including *E. coli* transformation and intergeneric conjugation between *E. coli*/*Streptomyces* were performed following standard protocols (Kieser et al., 2000; Green and Sambrook, 2012; Rebets et al., 2017). Plasmid DNA was purified with the BACMAX™ DNA purification kit (Lucigen, Middleton, WI, USA). Restriction endonucleases were used for plasmid diagnostic test (New England Biolabs, Ipswich, MA, USA).

### Metabolite Extraction and Analysis

*Streptomyces albus* 4N24 as well as the control strains *Streptomyces albus* De14 and *Streptomyces albus* subsp. *chlorinus* were cultivated in 15 mL TSB medium for 24 h at 28°C. Main cultures containing 50 mL of DNPM were inoculated with 1 mL of pre-culture. After 7 days of cultivation at 28°C, the secreted metabolites were extracted with ethyl acetate and butanol, followed by solvent evaporation. The dry extracts were solved in 1 mL methanol and 1 μL of the solved sample was separated using a Dionex Ultimate 3000 UPLC (Thermo Fisher Scientific, Waltham, MA, USA), and a 10-cm ACQUITY UPLC® BEH C18 column, 1.7 μm (Waters, Milford, MA, USA). The mobile phase was comprised of two solvents: formic acid solved in acetonitrile (0.1%) and formic acid solved in water (0.1%). Solvent concentrations varied in a linear gradient from 5 to 95% in 18 min at a flow rate of 0.6 mL/min. The UPLC system was coupled either to amaZon speed mass spectrometer or maXis high-resolution LC-QTOF system (Bruker, USA), allowing the mass spectrometry analysis of the extracts. The software Bruker Compass Data Analysis version 4.1 (Bruker, Billerica, MA, USA) was used for data analysis. Monoisotopic mass was searched in the natural product database DNP (Dictionary of Natural Products; Buckingham, 1993).

### Benzanthric Acid Isolation and Nuclear Magnetic Resonance (NMR) Spectroscopy

A flask containing 30 mL TSB medium was inoculated with *S. albus* 4N24 and incubated at 28°C for 24 h. The production culture consisted of 10 L divided into 100 flasks, each containing 100 mL DNPM medium and inoculated with 1 mL of pre-culture. After 7 days of cultivation at 28°C, metabolite extraction was performed as described above. The crude extract was fractionated by size-exclusion chromatography on an LH 20 Sephadex column (Sigma-Aldrich, USA) using methanol as the mobile phase. Resulting fractions were analyzed by LC-MS and those containing benzanthric acid were further separated by preparative HPLC (Waters 2545 Binary Gradient module, Waters, Milford, MA, USA) using a Nucleodur® C18 HTec column (5 μm, 250 × 21 mm, Macherey-Nagel, Düren, Germany) with a linear gradient of 0.1% formic acid solution in methanol against 0.1% formic acid solution in water, yielding 5 mg of benzanthric acid. UV spectra were recorded with a PAD detector (Photodiode Array Detector, Waters 2998, Waters, Milford, MA, USA). All reported NMR spectra were recorded at 298 K on a Bruker Avance 500 with a 5 mm BBO probe (Bruker, BioSpin GmbH, Rheinstetten, Germany) using DMSO-d_6_ (deuterated dimethyl sulfoxide) as solvent. The solvent peak was used as an internal standard and set to δ_H_ 2.49 for the ^1^H-NMR and δ_C_ 39.50 for the ^13^C-NMR, respectively. For the structural elucidation the following spectra were recorded with standard pulse programs: ^1^H-NMR, ^13^C-NMR, ^1^H-^1^H-correlated spectroscopy (COZY), heteronuclear single quantum spectroscopy (HSQC), heteronuclear multiple bond correlation (HMBC), and rotating frame Overhauser enhancement spectroscopy (ROESY, spin-lock: 300 ms).

### Feeding Experiment With Anthranilic Acid (Phenyl-^13^C_6_)

Two flasks containing 25 mL of DNPM medium were inoculated with *S. albus* 4N24 as described above (Section Metabolite Extraction and Analysis). One of the cultures was supplemented with 5 mg/mL of anthranilic acid (phenyl-^13^C_6_) (Cambridge Isotope Laboratories, Andover, MA, USA) at intervals of 12 h for 4 days, while the second one was used as a control. After further 24 h cultivation, the metabolites were extracted from the supernatant as described in Section Metabolite Extraction and Analysis.

### Antimicrobial Susceptibility Test

Susceptibility tests were performed by the disk diffusion method described in Bauer et al. (1966). Ten mL of LB soft agar (10 g/l tryptone, 10 g/l NaCl, 5 g/l yeast extract, 7 g/l agar) were inoculated with the strains *Escherichia coli* GB2005, *Bacillus subtilis* ATCC 6633 or *Pseudomonas putida* KT2440 and poured on LB agar plates. Four paper disks (Macherey and Nagel, Düren, Germany) were coated with 100, 50, 10, and 0.5 μg of benzanthric acid solved in DMSO, respectively, and placed onto the solidified soft agar. DMSO was used as a negative control and the antibiotics ampicillin, chloramphenicol, and nalidixic acid (50 mg/mL, respectively) as positive controls. The plates were incubated at 28°C overnight.

### Herbicidal Pre-emergence Test

Seeds of *Agrostis stolonifera* (Juliwa HESA, Heidelberg, Germany) were placed into the wells of a 96-well microtiter plate (Sarstedt, Nümbrecht, Germany). A solution containing 2.2 g/l Murashige & Skoog plant salts (Serva, Heidelberg, Germany) and 1.6 g/l Gamborg's B5 plant medium (Serva, Heidelberg, Germany) was added to the wells. Decreasing concentrations of benzanthric acid solved in DMSO were added (2 mM, 1 mM, 0.5 mM, 0.25 mM, 125 μM, and 62.5 μM). Identical volumes of DMSO without benzanthric acid were used as a toxicity test of the organic solvent. The solution containing the plant medium was used as a negative control. The plate was closed and incubated at room temperature under constant light (Osram Fluora lamp) in a humidity chamber. After 3 days of incubation, the plate lid was removed and a container with tap water was placed inside the chamber for increasing the air humidity. The plate was incubated up to 6 days. Three technical replicates were performed.

### Genome Mining and Bioinformatics Analysis

The online tool antiSMASH (https://antismash.secondarymetabolites.org/#!/start) was used for the identification of secondary metabolite biosynthetic gene clusters in the genome of *S. albus* subsp. *chlorinus* (Weber et al., 2015). Gene cluster analysis was performed with the help of the software Geneious 11.0.3 (Kearse et al., 2012).

## Results

**Figure 1:**
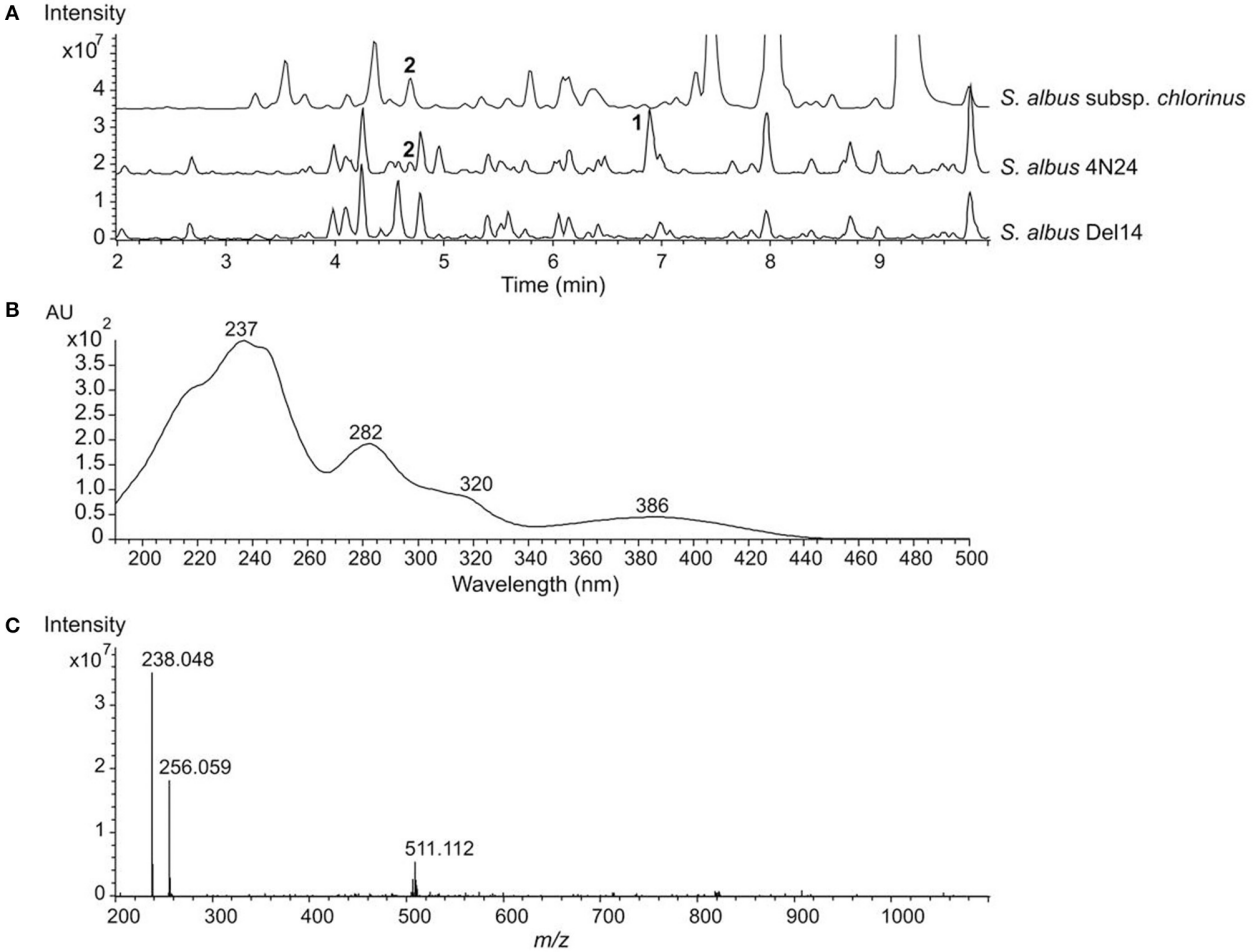
HPLC-MS analysis of benzanthric acid production. **(A)** Base peak chromatograms of crude extracts from the parent strain *S. albus* subsp. *chlorinus*, the recombinant strain *S. albus* 4N24 harboring the nybomycin gene cluster and the host strain *S. albus* Del14. Peaks corresponding to benzanthric acid and nybomycin are indicated by **1** and **2**, respectively. **(B)** UV spectrum of benzanthric acid. **(C)** Mass spectrum of benzanthric acid (*m/z* 256.0 [M+H]^+^). The signal at *m/z* 238.0 corresponds to the [M+H-H_2_O]^+^ ion and the signal at *m/z* 511.1 corresponds to the [2M+H]^+^ ion.

We have previously reported the identification of the nybomycin gene cluster from *S. albus* subsp. *chlorinus* NRRL B-24108 through heterologous expression of BAC 4N24 harboring the *nyb* genes in the host *S. albus* Del14 (Rodriguez Estevez et al., 2018). In addition to nybomycin, the HPLC-MS analysis of an extract from the recombinant strain *S. albus* 4N24 expressing the *nyb* genes revealed the presence of a peak at t_R_ = 6.9 min and *m/z* 256.059 [M + H]^+^ (Figure 1), exhibiting UV absorption signals at λ_max_ 237, 282, 320, and 386 nm (Figure 1B). Unlike nybomycin, which is produced by both *S. albus* 4N24 and the parent strain *S. albus* subsp. *chlorinus*, the peak at *m/z* 256.059 was solely detected in the extract of *S. albus* 4N24 (Figure 1). The search of the monoisotopic mass 255.051 in a natural product database yielded no coincidences, which suggested a putatively new compound and encouraged us to purify it for structure elucidation.

**Figure 2:**
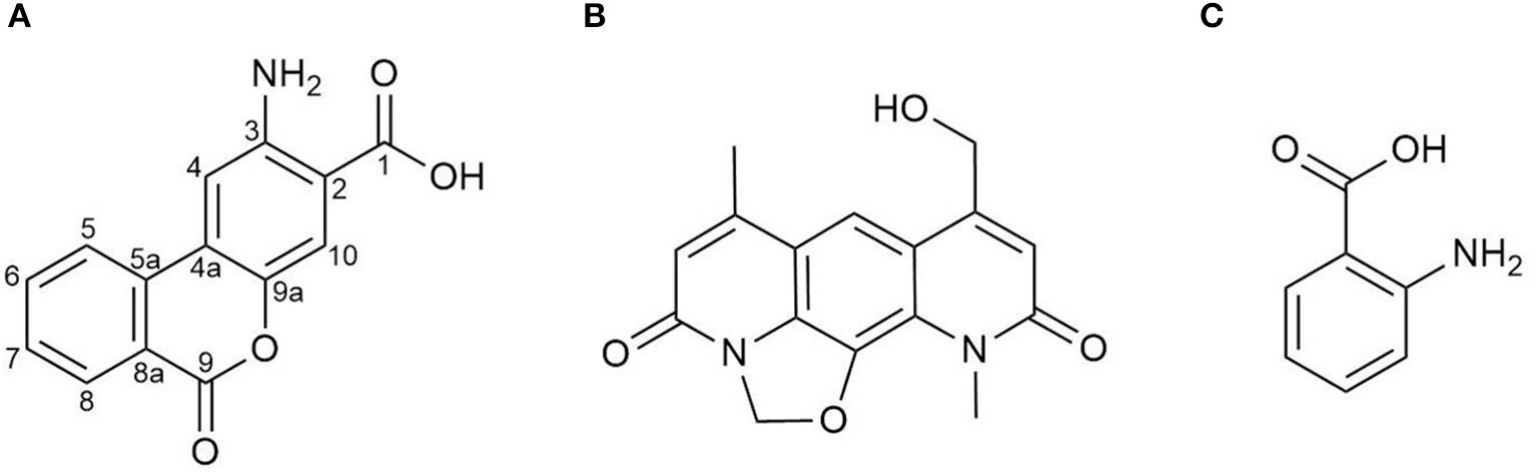
Chemical structures of **(A)** benzanthric acid, **(B)** nybomycin and **(C)** anthranilic acid.

After seven days of cultivation of the strain *S. albus* 4N24 in 10 L of DNPM liquid medium, the broth was centrifuged and the metabolites were extracted from the supernatant. The extract was fractionated by size-exclusion chromatography through a Sephadex column followed by preparative high performance liquid chromatography (HPLC) to yield 5 mg of the novel metabolite benzanthric acid (Figure 2A). The molecular formula of benzanthric acid was determined as C_14_H_9_NO_4_ by high-resolution electrospray ionization mass spectrometry (HRESMS) and NMR (*m/z* 256.05949, −3.688 ppm). The analysis of the ^13^C-NMR (125 MHz, DMSO-d_6_) revealed the presence of fourteen carbons. Two carbons were assigned as carbonyl groups (δ_C_ 168.67 C-1, 160.22 C-9). The twelve remaining carbons were assigned as olefinic carbons (δ_C_ 135.28 C-6, 133.40 C-5a, 129.95[2x] C7 & C8, 122.58 C-5, 122.34 C-4a, 121.52 C-8a, 118.57 C-10, 113.29 C-2, 108.84 C-4) including one oxygenated carbon (δ_C_ 140.59 C-9a) and one aminated carbon (δ_C_ 147.81 C-3; Supplementary Figures 1–3). The ^1^H-NMR (500 MHz, DMSO-d_6_) showed six aromatic signals: δ_H_ 8.24 (1H, dd; J_1, 2_ = 7.9, 1.1 Hz; H-8), δ_H_ 8.16 (1H, d, J = 8.1 Hz, H-5), δ_H_ 7.96 (1H, dt, J_1, 2_ = 7.7, 1.1 Hz, H-6), δ_H_ 7.70 (1H, dt, J_1, 2_ = 7.6, 0.6 Hz, H-7), δ_H_ 7.64 (1H, s, H-10), and δ_H_ 7.57 (1H, s, H-4; Supplementary Figures 1, 4–6). The ^1^H-^1^H-COZY spectrum showed correlations between H-5 and H-6, H-6 and H-7, H-7, and H-8 (Supplementary Figures 7, 8). The phase sensitive HSQC spectrum revealed eight quaternary carbons (C-1, C-2, C-3, C-4a, C-5a, C-8a, C-9, and C-9a; Supplementary Figures 9, 10). Through correlation in the heteronuclear multiple bond correlation (HMBC) experiment connections between the spin systems were identified (Supplementary Figures 11, 12). The complete list including all correlations can be found in Table 1. Important correlations to establish connections between the spin systems were H-4 to C-5a, H5 to C-4a as well as H-10 to C-4a and C-4. A ^15^N-HMBC experiment revealed the correlation between H-4 and the nitrogen of the primary aromatic amine (Supplementary Figure 13). The ROESY spectrum showed a correlation through space between H-4 and H-5 (Supplementary Figure 14).

**Table 1:** NMR chemical shifts and 2D correlations of benzanthric acid.

**Position**	**δ^13^C[ppm]^a^ type**	**δ^1^H[ppm] ^b^ (J in Hz)**	**COZY**	**HMBC^c^**
1	168.67 C	–	–	–
2	113.29 C	–	–	–
3	147.81 C	–	–	–
4	108.84 CH	7.57 s	–	C-1, C-2, C-3, C-5a, C-9a, C-10
4a	122.34 C	–	–	–
5a	133.40 C	–	–	–
5	122.58 CH	8.16 d (8.1)	6	C-4a, C-5a, C-6, C-8, C-8a, C-9
6	135.28 CH	7.96 dt (7.7, 1.1)	5; 7	C-5a, C-7, C-8
7	129.95 CH	7.70 dt (7.6, 0.6)	6; 8	C-5, C-5a, C-6, C-8, C-8a, C-9
8	129.95 CH	8.24 dd (7.9, 1.1)	7	C-5a, C-6, C-9
8a	121.52 C	–	–	–
9	160.22 C	–	–	–
9a	140.59 C	–	–	–
10	118.57 CH	7.64 s	–	C-1, C-2, C-4, C-4a, C-5a, C-9a, C-10
COOH^d^	–	7.00–9.50 s br	–	–
${\text{NH}}_{2}^{\text{d}}$	–	7.00–9.50 s br	–	–

a *125 MHz for ^13^C-NMR*.

b *500 MHz for ^1^H-NMR*.

c *HMBC correlations from protons to the indicated carbons*.

d *Exchangeable protons*.

**Figure 3:**
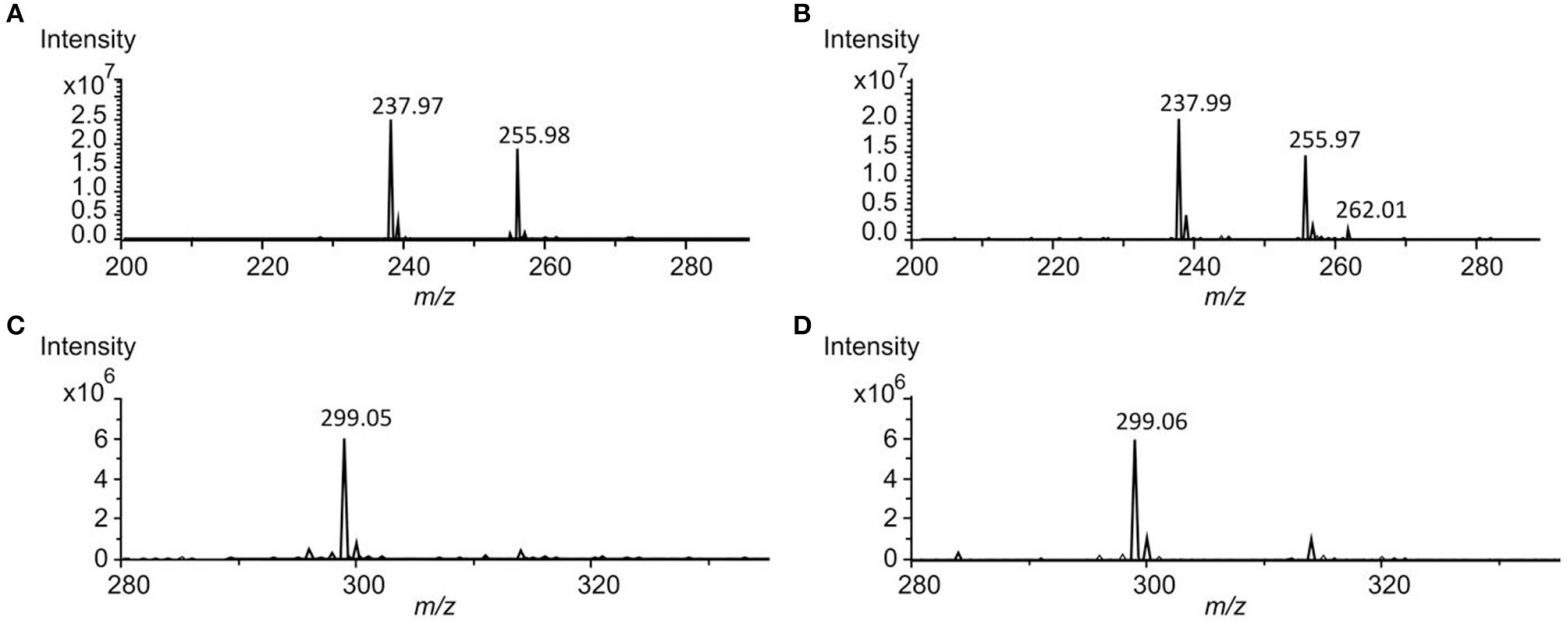
Analysis of anthranilic acid (phenyl-^13^C_6_) incorporation. **(A)** Mass spectrum of benzanthric acid (*m/z* 255.9 [M+H]^+^) extracted from the culture of *S. albus* 4N24. The signal at *m/z* 237.9 corresponds to the [M+H-H_2_O]^+^ ion. **(B)** Mass spectrum of benzanthric acid extracted from the culture of *S. albus* 4N24 fed with anthranilic acid (phenyl-^13^C_6_). The signal at *m/z* 262.0 corresponds to the [M+H]^+^ ion after incorporation of the labeled anthranilic acid. **(C)** Mass spectrum of nybomycin (*m/z* 299.0 [M+H]^+^) extracted from the culture of *S. albus* 4N24. **(D)** Mass spectrum of nybomycin extracted from the culture of *S. albus* 4N24 fed with anthranilic acid (phenyl-^13^C_6_). No incorporation of anthranilic acid can be detected.

Benzanthric acid's structure has been determined as a benzoate part bound to an anthranilate moiety. This arrangement considerably differs from the core structure of nybomycin (Figures 2A,B), which is also produced by the strain *S. albus* 4N24 harboring the *nyb* genes. In order to test whether anthranilic acid functions as a precursor in the biosynthesis of benzanthric acid and nybomycin, a culture of *S. albus* 4N24 was fed with anthranilic acid (phenyl-^13^C_6_) (Figure 2C). HPLC-MS analysis of the resulting extract revealed the incorporation of the labeled anthranilate into the structure of benzanthric acid. The mass spectrum of benzanthric acid shows two signals: a signal at *m/z* 256 corresponding to the [M+H]^+^ ion of the compound and a signal at *m/z* 238 corresponding to its derivative after water loss (Figure 3A). After feeding with labeled anthranilic acid an additional signal at *m/z* 262 is observed (Figure 3B), implying the incorporation of the six ^13^C atoms of the labeled anthranilate into benzanthric acid. No incorporation of anthranilic acid into the structure of nybomycin was detected (Figures 3C,D).

The antimicrobial activity of benzanthric acid against the Gram-negative bacteria *Escherichia coli* and *Pseudomonas putida*, as well as the Gram-positive bacterium *Bacillus subtilis* was assayed through disk diffusion test, revealing no growth inhibitory activity (Supplementary Figure 15A). The herbicidal activity of the new metabolite was tested against the monocot grass species *Agrostis stolonifera*. The purified benzanthric acid was added to the plant seeds and incubated in a minimal medium. Seed germination was observed after 3 days, indicating no inhibitory effect against the tested plant (Supplementary Figure 15B).

## Discussion

In this paper, we describe the isolation and chemical structure elucidation of the novel metabolite benzanthric acid, produced by *S. albus* 4N24 containing the nybomycin biosynthetic gene cluster. The strain simultaneously produces nybomycin and benzanthric acid, suggesting that both compounds might share common biosynthetic steps. The structure of benzanthric acid (Figure 2A) suggests anthranilate and benzoate as possible biosynthetic precursors. The precursor role of anthranilic acid has been proved by feeding studies. Interestingly, no incorporation of anthranilic acid into nybomycin's structure could be detected, indicating substantial differences in the biosynthetic routes leading to nybomycin and benzanthric acid.

In living organisms, anthranilic acid is mainly synthesized either through the shikimate pathway or through the tryptophan degradation pathway (Haslam, 1974; Kurnasov et al., 2003). One of the nybomycin biosynthetic genes, *nybF*, encodes a putative 3-Deoxy-D-arabinoheptulosonate 7-phosphate (DAHP) synthase, which catalyzes the first reaction of the shikimate pathway. Since the DAHP synthase controls the amount of carbon entering the pathway, the expression of the *nybF* gene can lead to its upregulation and increased intracellular concentrations of benzanthric acid's precursor—anthranilic acid. Additionally, the product of the *nybD* gene shows homology at the protein level with anthranilate synthase and might be also responsible for the additional supply of anthranilic acid. In this case, anthranilic acid is most probably only a by-product of the enzyme's reaction, since it was shown not to be a precursor of nybomycin biosynthesis.

We propose that benzoic acid serves as the second direct precursor for benzanthric acid production. No genes which could lead to the biosynthesis of benzoic acid were identified within the DNA fragment containing the nybomycin cluster. Therefore, it is likely that benzoic acid is provided by the metabolism of the host strain *S. albus* Del14. The phenylalanine degradation pathway could be responsible for the supply of this precursor (Tabor and Tabor, 1970).

The attachment of benzoic acid to the anthranilate moiety is necessary for the formation of benzanthric acid. The enzyme catalyzing this biosynthetic step could not be identified within the nybomycin gene cluster. We propose that the required enzyme is encoded by the genome of the host strain *S. albus* Del14. This is further supported by the data revealing no production of benzanthric acid by the original nybomycin producer (Figure 1A).

The fact that the new compound, benzanthric acid, could be generated through the expression of the characterized nybomycin gene cluster in the well-studied heterologous host *S. albus* Del14 is intriguing. The isolated compound can be found neither in the extracts of the natural nybomycin producer nor in that of the heterologous host *S. albus* Del14. Benzanthric acid is also not a degradation product of nybomycin. The most plausible explanation for the origin of the isolated compound is the interplay between the host's metabolism and the introduced biosynthetic pathway. The isolation of benzanthric acid raises the question of whether this is rather an exception or the integration of foreign metabolic pathways or their parts into the host's metabolism can be used as a tool for the generation of new natural products.

## Data Availability Statement

The datasets generated for this study can be found in the GenBank under accession number MH924838.

## Author Contributions

MR, MM, and AL designed the experiments. MR performed the experiments. NG and JZ performed and evaluated the NMR analysis. MR, NG, and MM wrote the manuscript. All the authors reviewed the manuscript.

### Conflict of Interest

The authors declare that the research was conducted in the absence of any commercial or financial relationships that could be construed as a potential conflict of interest.
