# Supplementary material for: Benzanthric Acid, a Novel Metabolite From Streptomyces albus Del14 Expressing the Nybomycin Gene Cluster
Source: Front Chem. 2020 Jan 10;7:896. doi: 10.3389/fchem.2019.00896 (PMC6965495; doi:10.3389/fchem.2019.00896)
Supplement: Supplementary file 1 [file Table_1.DOCX]

Supplementary Material

# Supplementary Tables

Supplementary Table 1. Bacterial strains and BAC (bacterial artificial chromosome) vectors used in this work.

| **Bacterial strain** | **Features** | **Reference/ Source** |
| --- | --- | --- |
| *Streptomyces* *albus* subsp. *chlorinus* NRRL B-24108 | *S. albus* subspecies strain harboring nybomycin biosynthetic gene cluster | (Hahn et al. 2009) |
| *Streptomyces albus* Del14 | Wild-type strain | (Myronovskyi et al. 2018) |
| *Streptomyces albus* 4N24 | *S. albus* strain with BAC 4N24 insertion | (Rodriguez Estevez et al. 2018) |
| *Escherichia coli* ET12567 pUB307 | Donor strain for  intergeneric conjugation | (Flett et al. 1997) |
| *Escherichia coli* DH10β | General cloning strain | (Grant et al. 1990) |
| **BACs** |  |  |
| pSMART | AmR; BAC vector | Lucigen (USA) |
| 4N24 | BAC containing nybomycin  gene cluster | Intact Genomics (USA) |

# Supplementary Figures

Supplementary Figure 1. Structure of benzanthric acid including chemical shifts of protons and carbons.


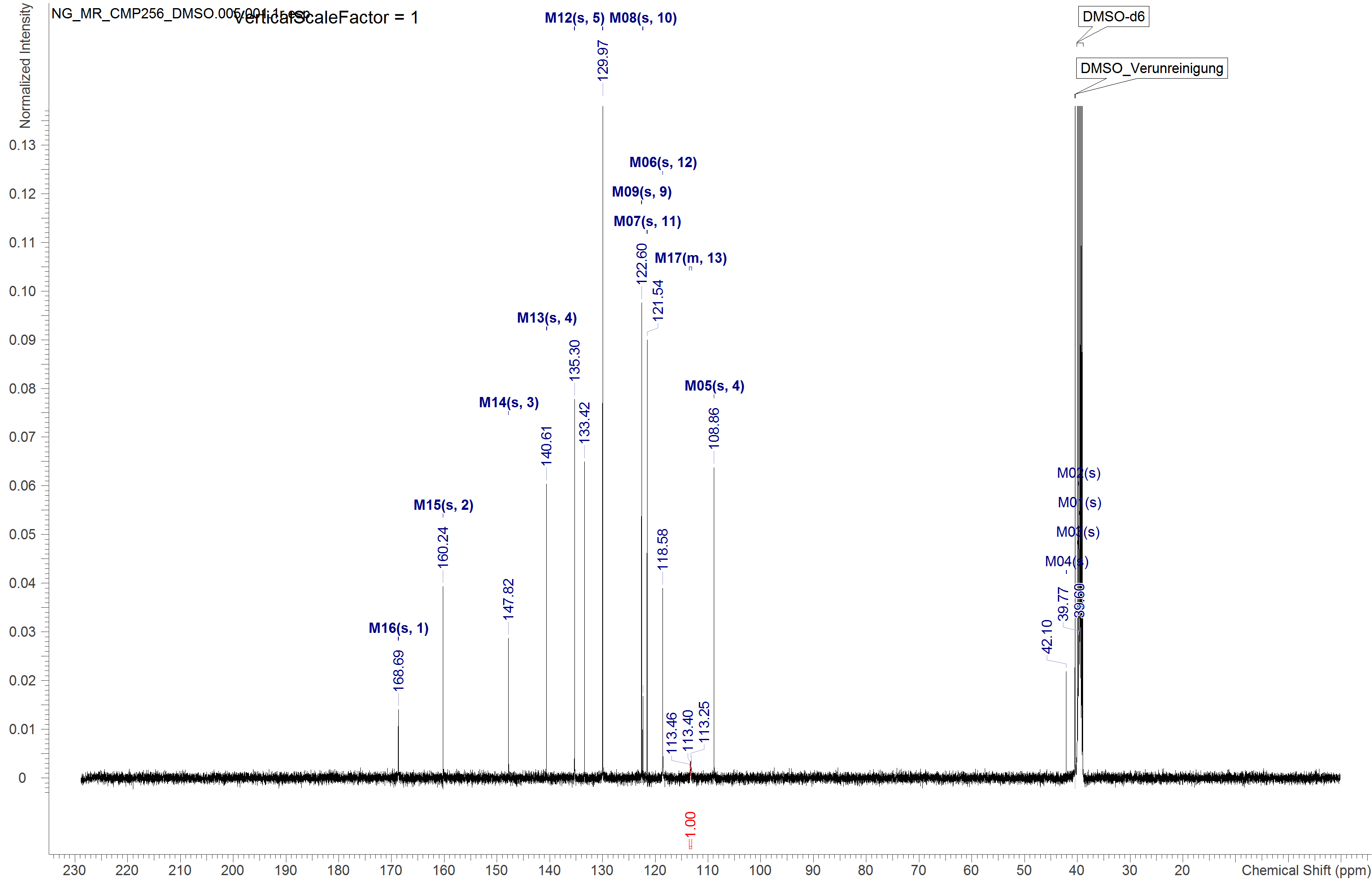


**Supplementary Figure 2.** ^13^C-NMR spectrum (125 MHz, DMSO-d_6_) of benzanthric acid.


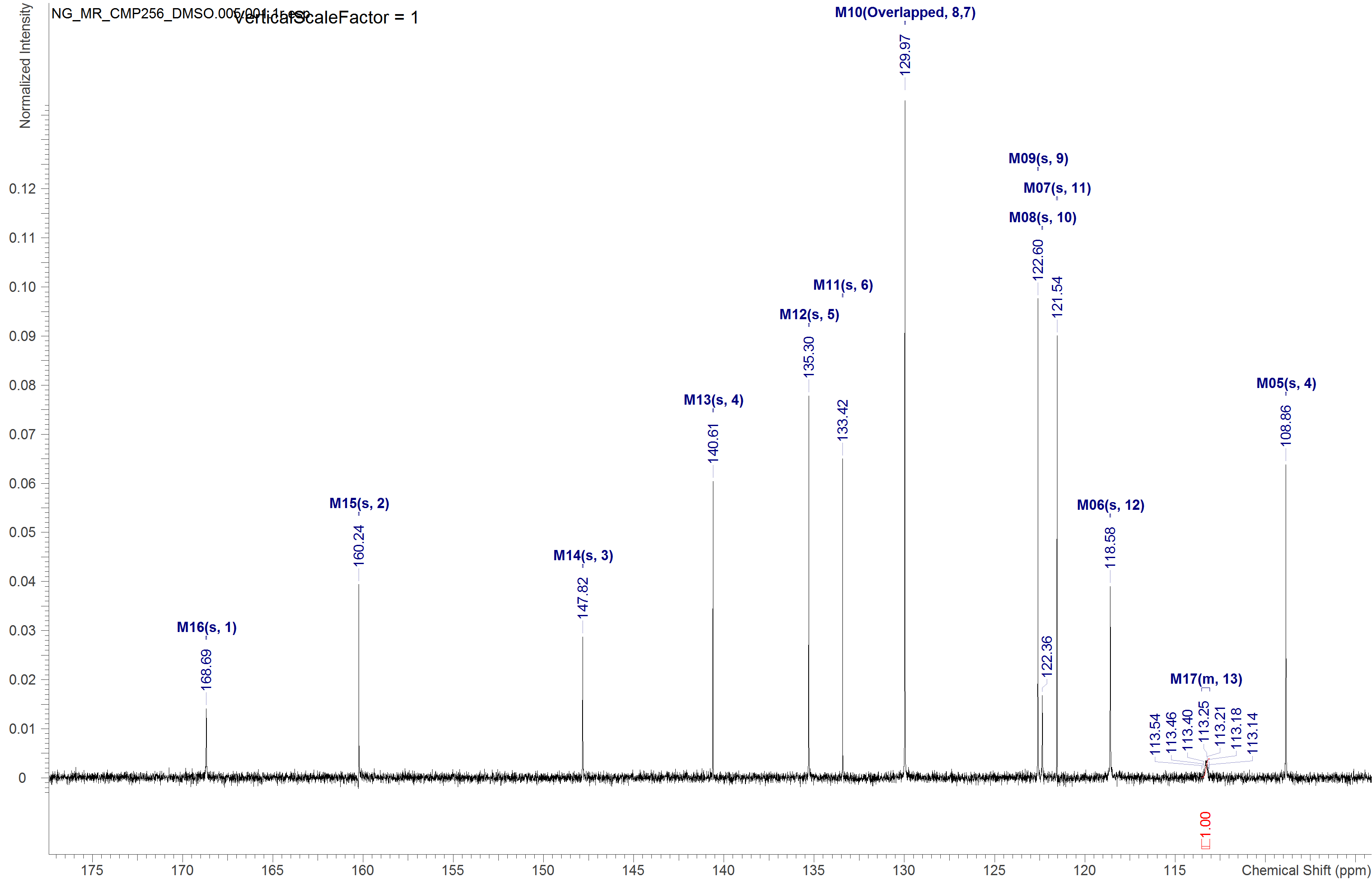


Supplementary Figure 3. ^13^C-NMR spectrum (125 MHz, DMSO-d_6_) of benzanthric acid (100 ppm – 175 ppm).


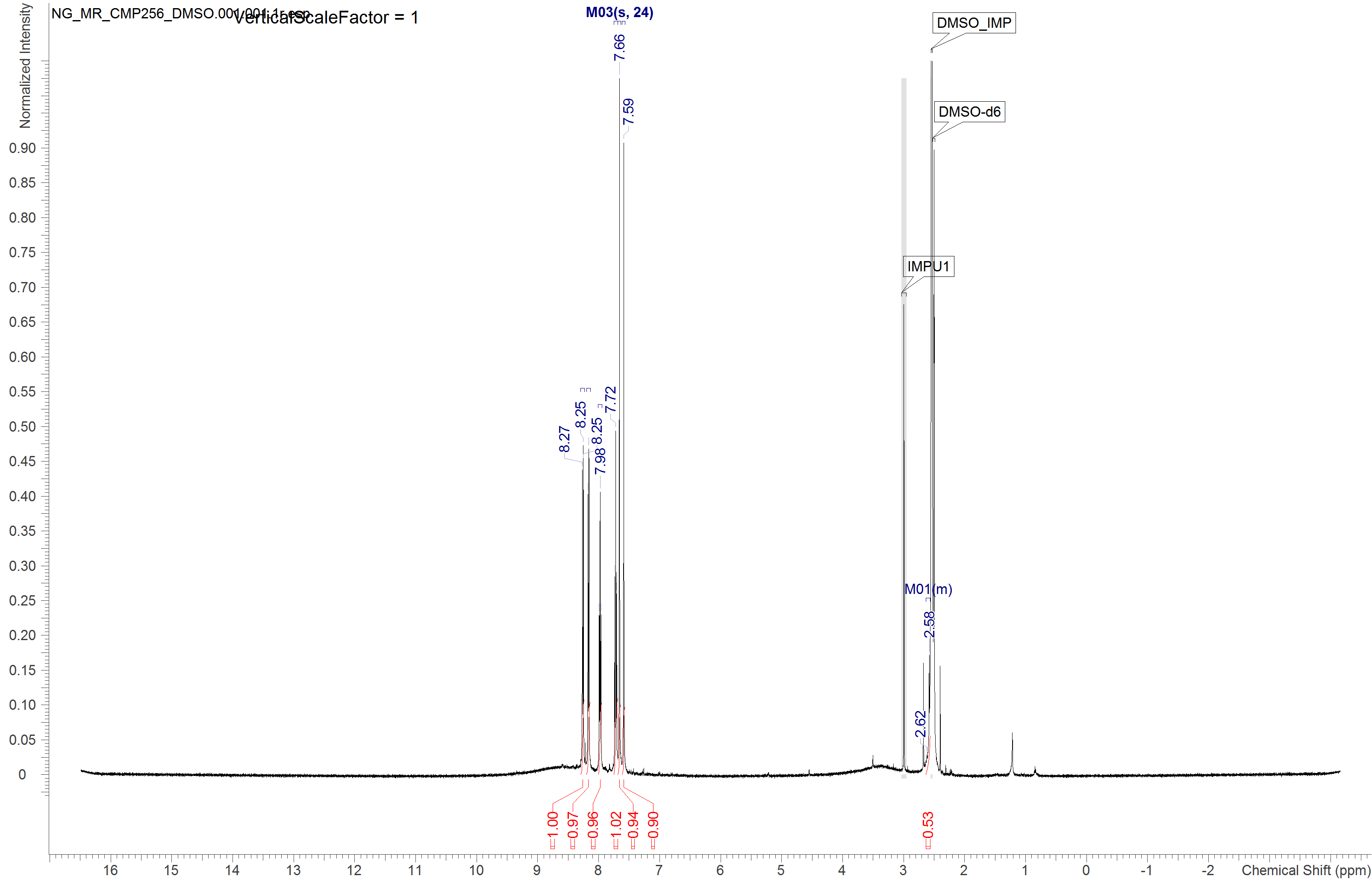


Supplementary Figure 4. ^1^H-NMR spectrum (500 MHz, DMSO-d_6_) of benzanthric acid.


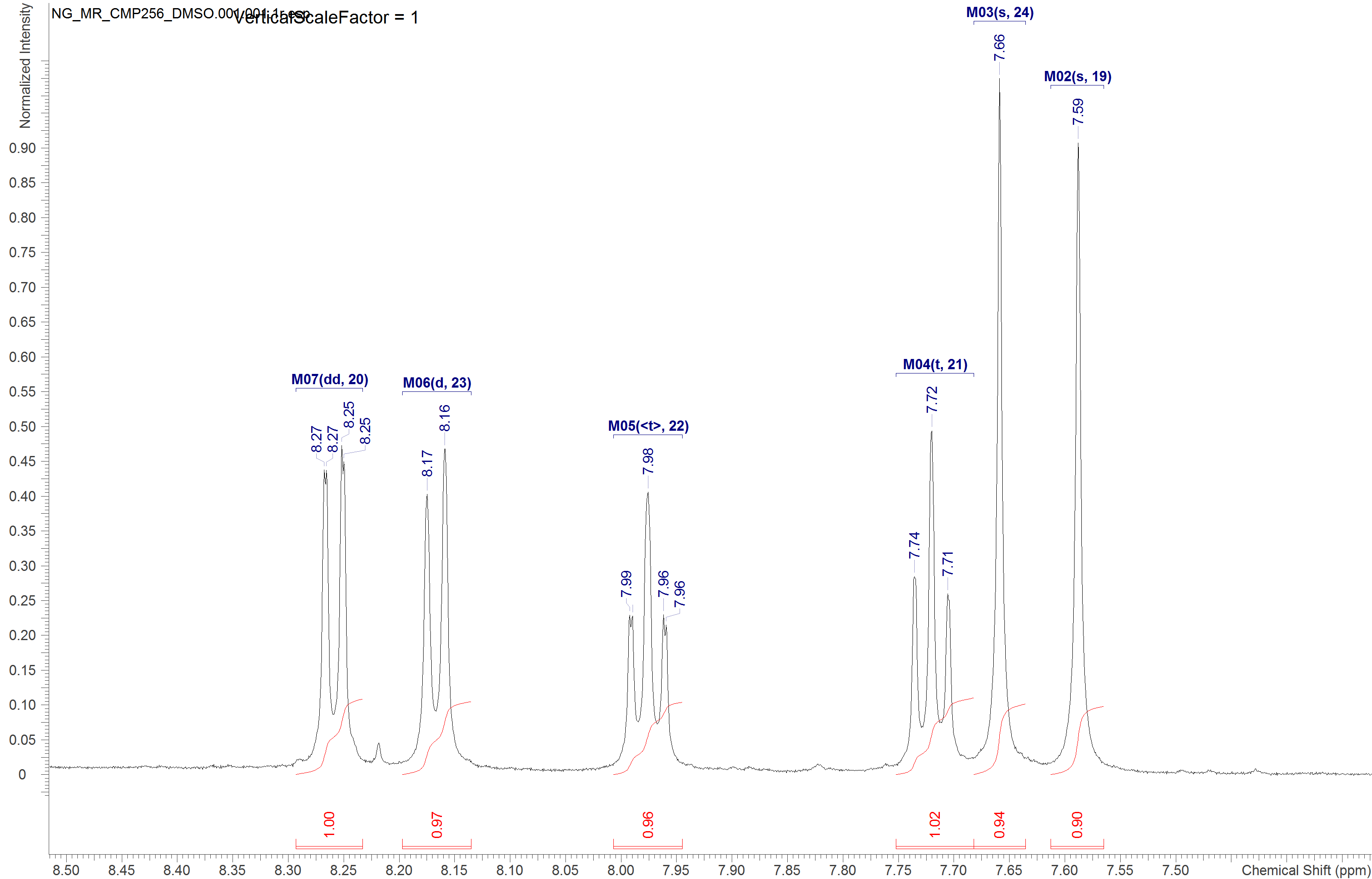


Supplementary Figure 5. ^1^H-NMR spectrum (500 MHz, DMSO-d_6_) of benzanthric acid (7.5 ppm – 8.5 ppm).


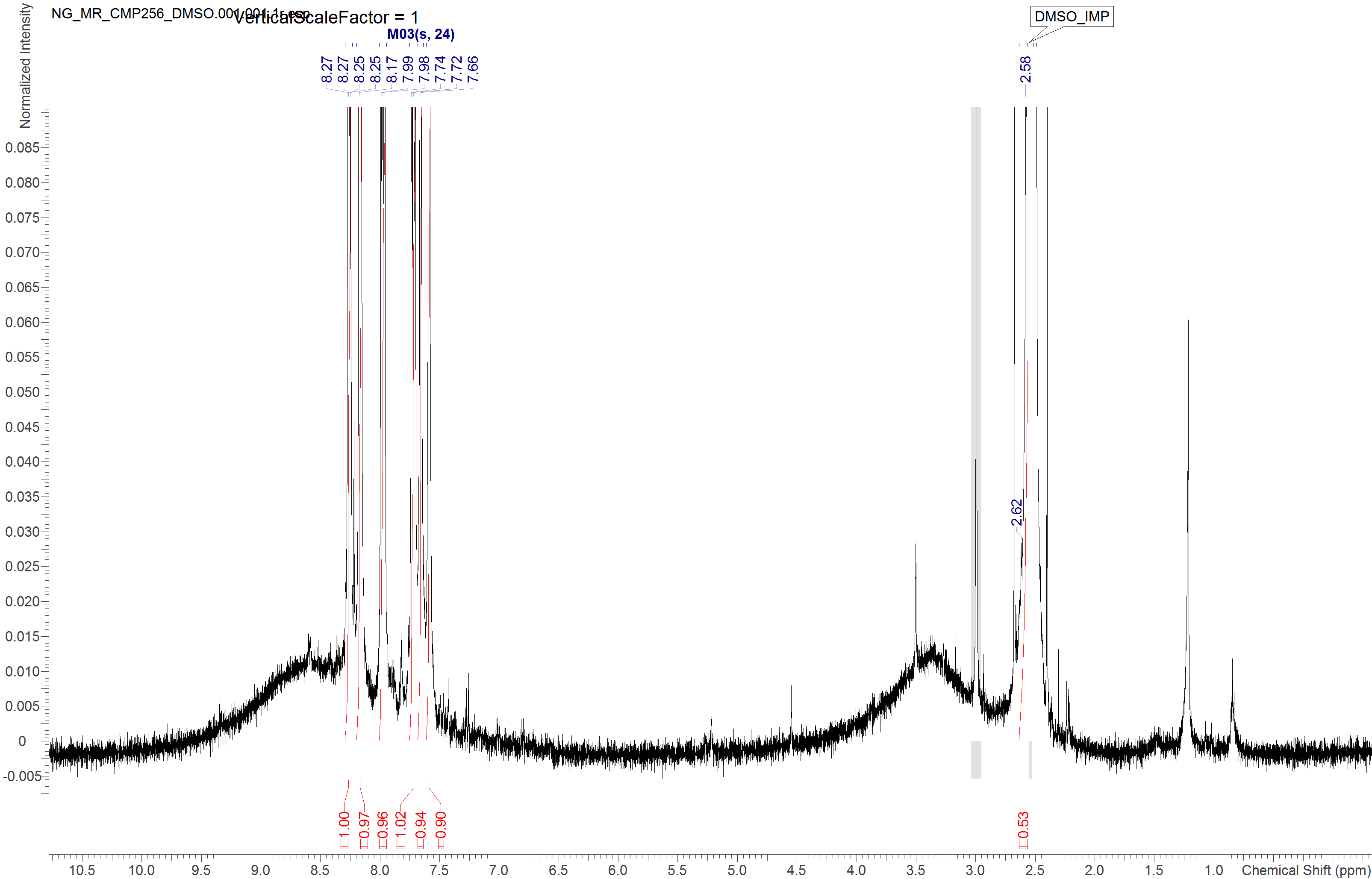


Supplementary Figure 6. ^1^H-NMR spectrum (500 MHz, DMSO-d_6_) of benzanthric acid with increased intensity.

**
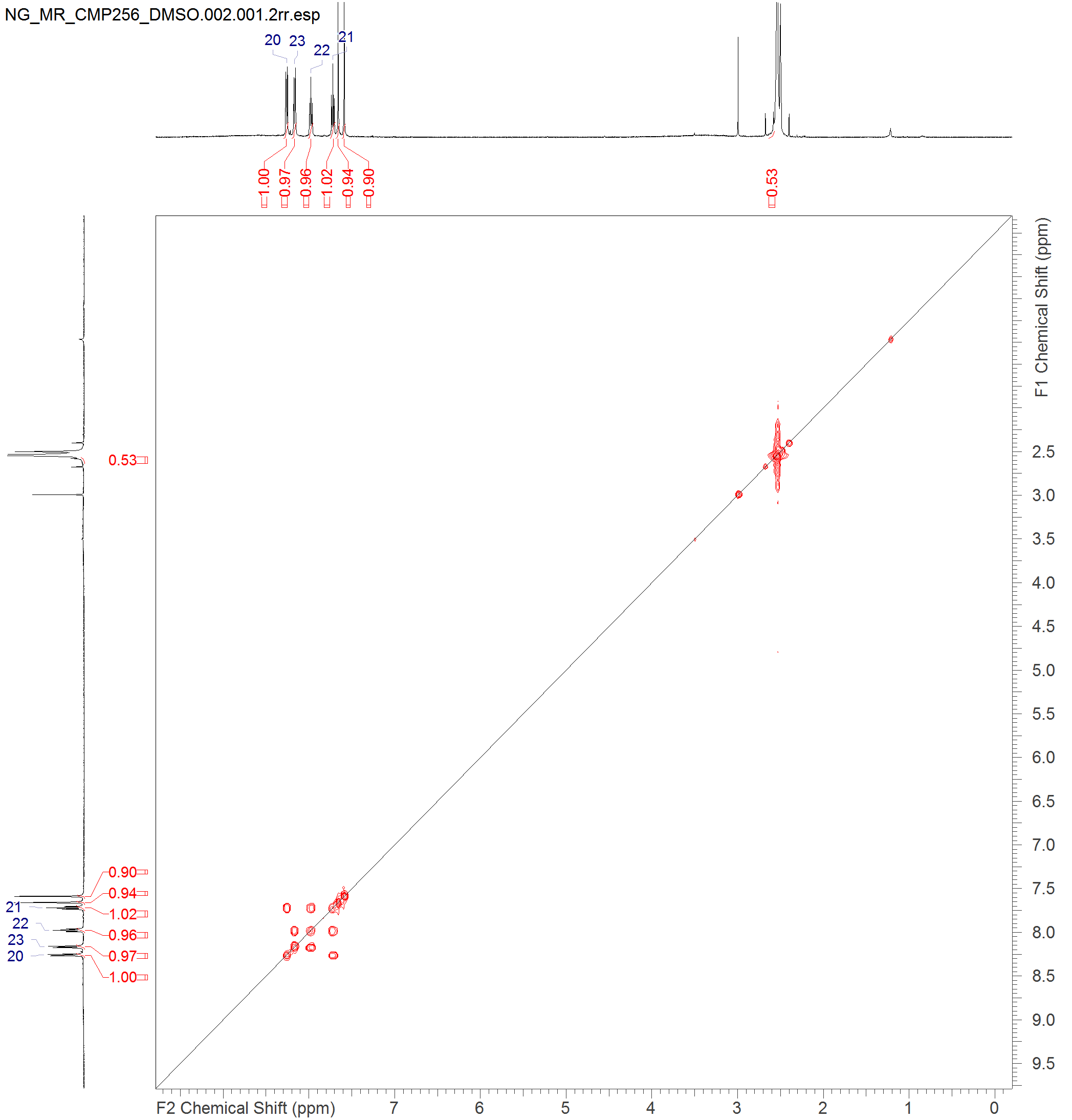
**

**Supplementary Figure 7.** ^1^H-^1^H-COSY spectrum (DMSO-d_6_) of benzanthric acid.

**
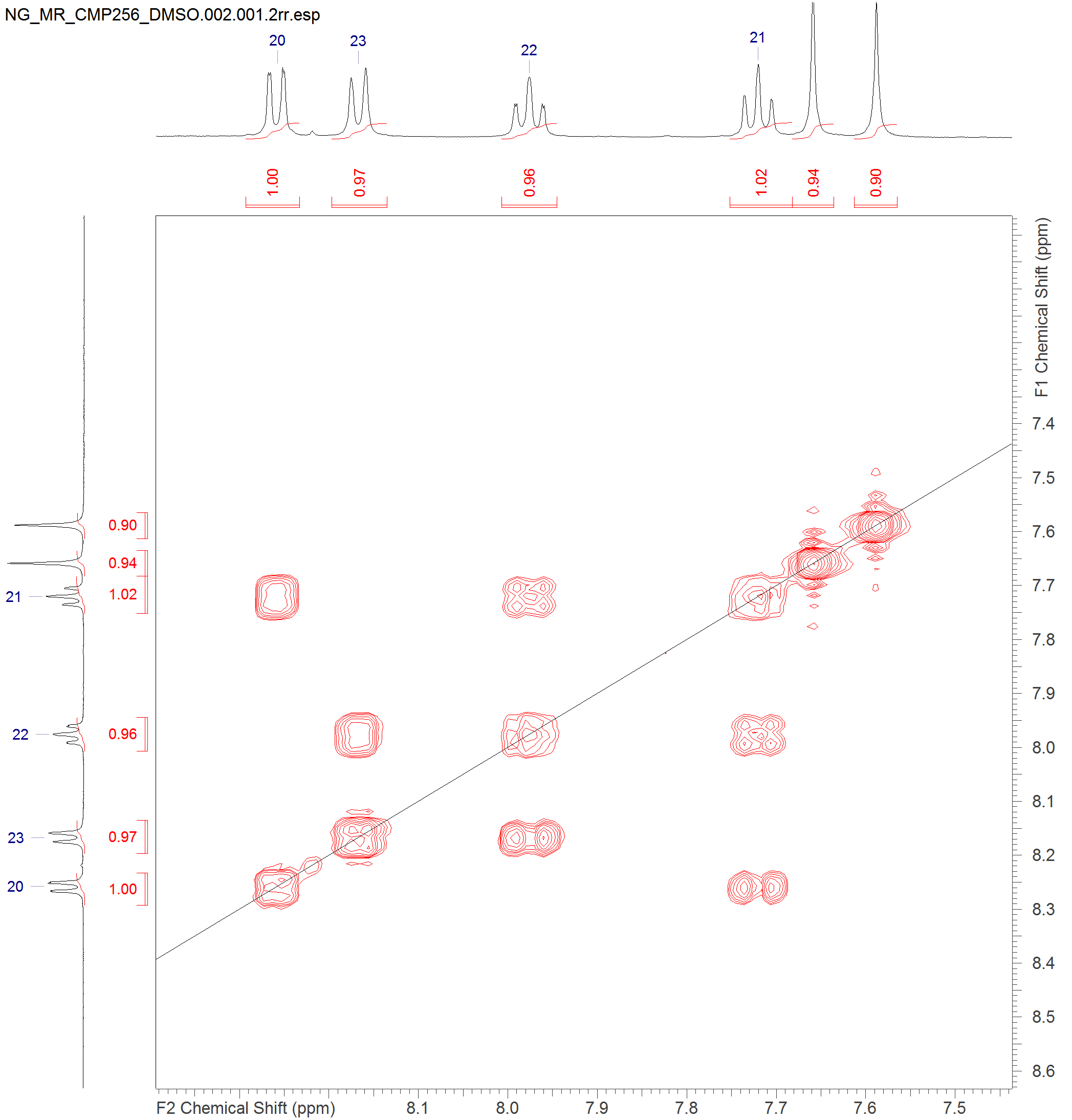
**

Supplementary Figure 8 ^1^H-^1^H-COSY spectrum (DMSO-d_6_) of benzanthric acid (7.2 ppm – 8.6 ppm).

**
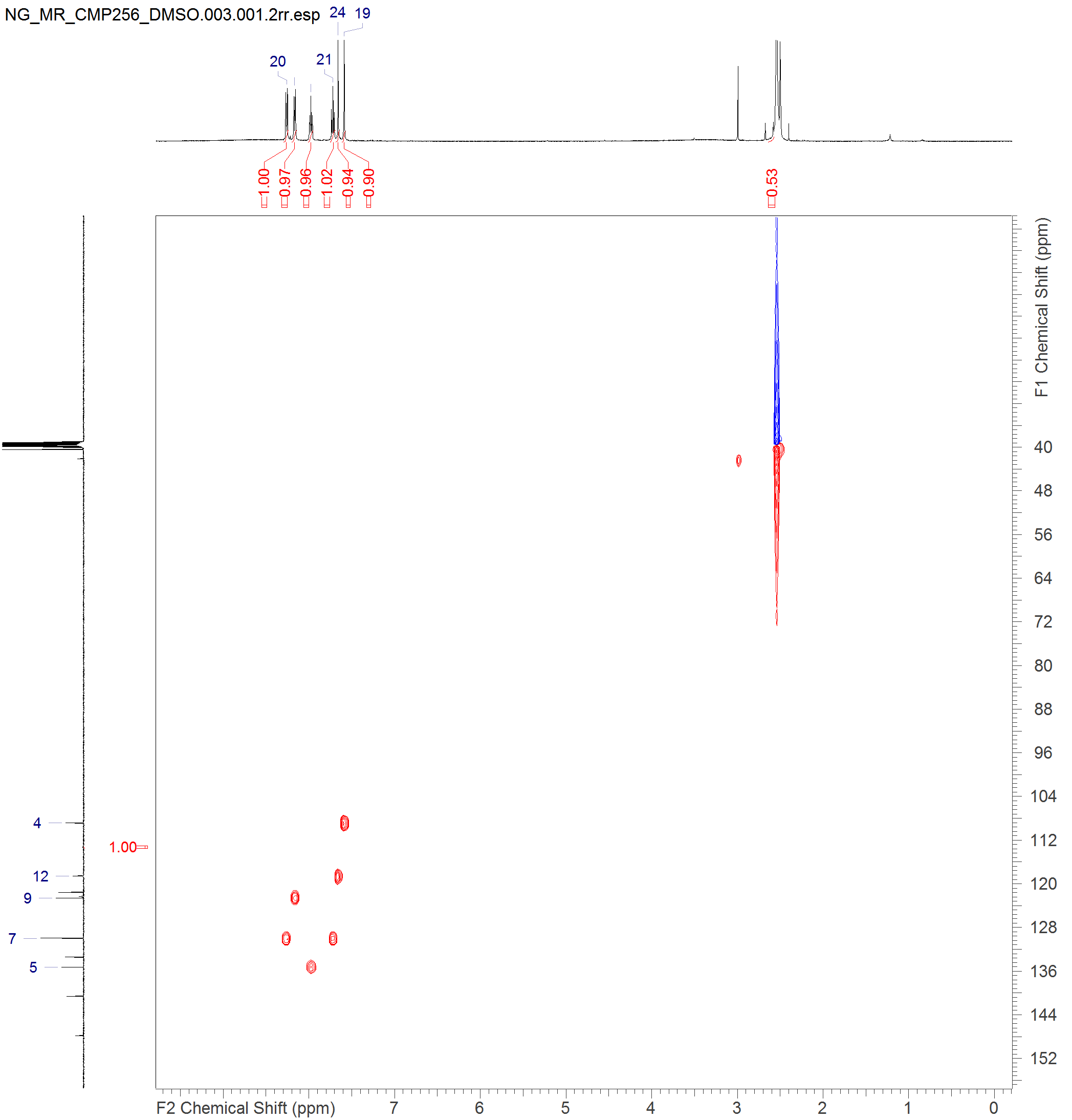
**

Supplementary Figure 9. HSQC spectrum (DMSO-d_6_) of benzanthric acid.


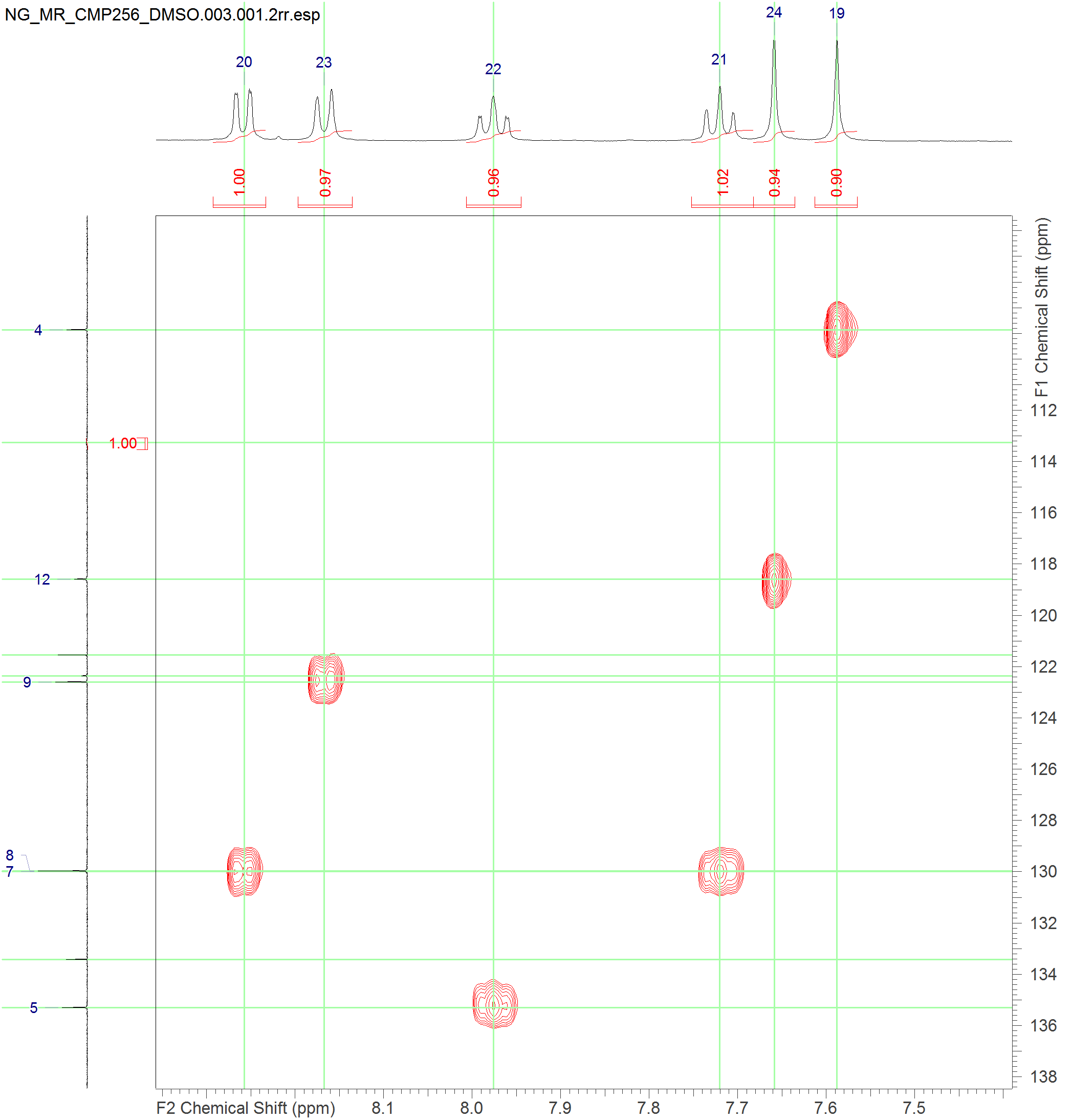


Supplementary Figure 10. HSQC spectrum (DMSO-d_6_) of benzanthric acid (7.5 ppm – 8.3 ppm & 105 ppm – 138 ppm).


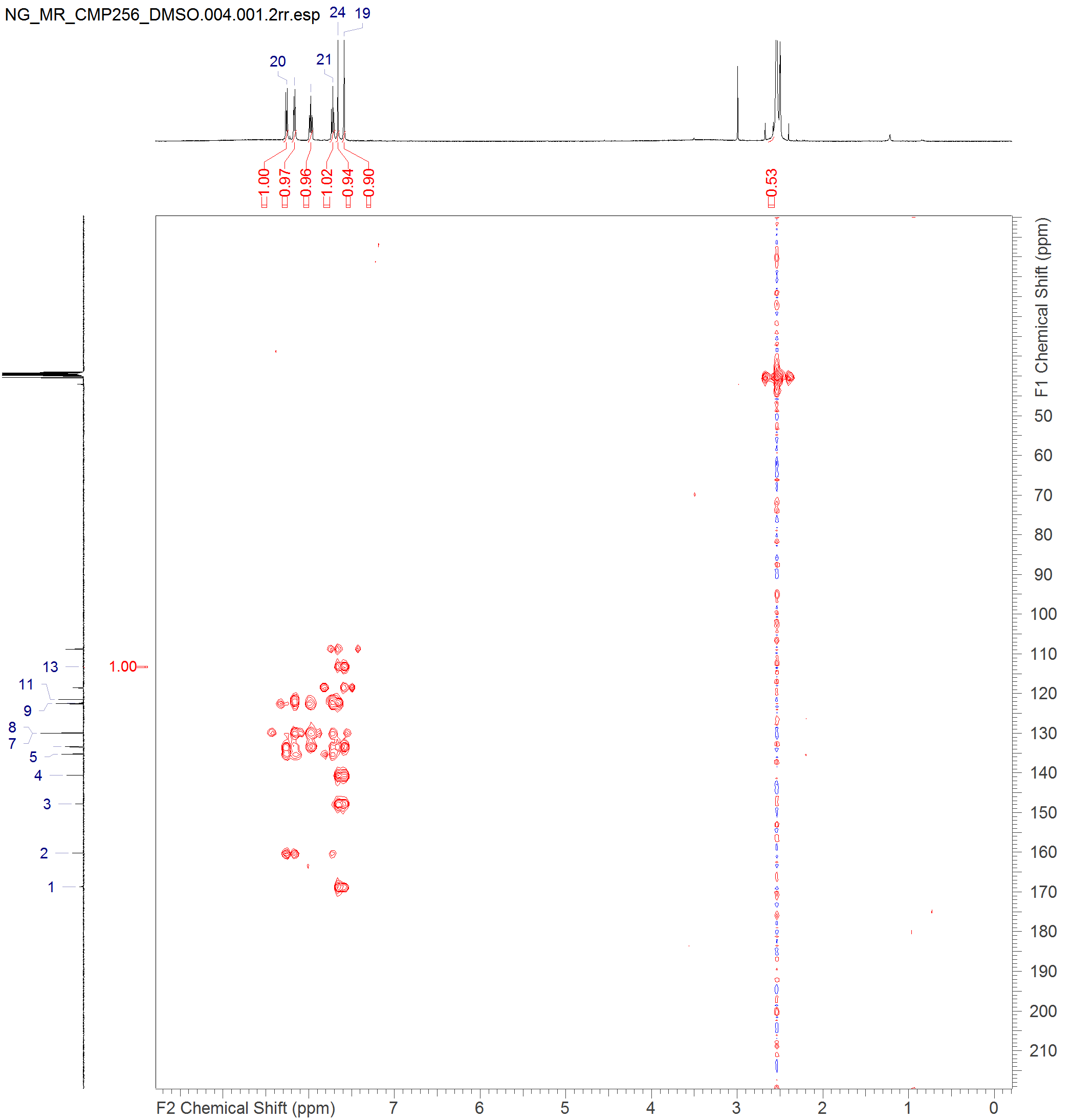


Supplementary Figure 11. HMBC spectrum (DMSO-d_6_) of benzanthric acid (7.5 ppm – 8.3 ppm & 105 ppm – 138 ppm).


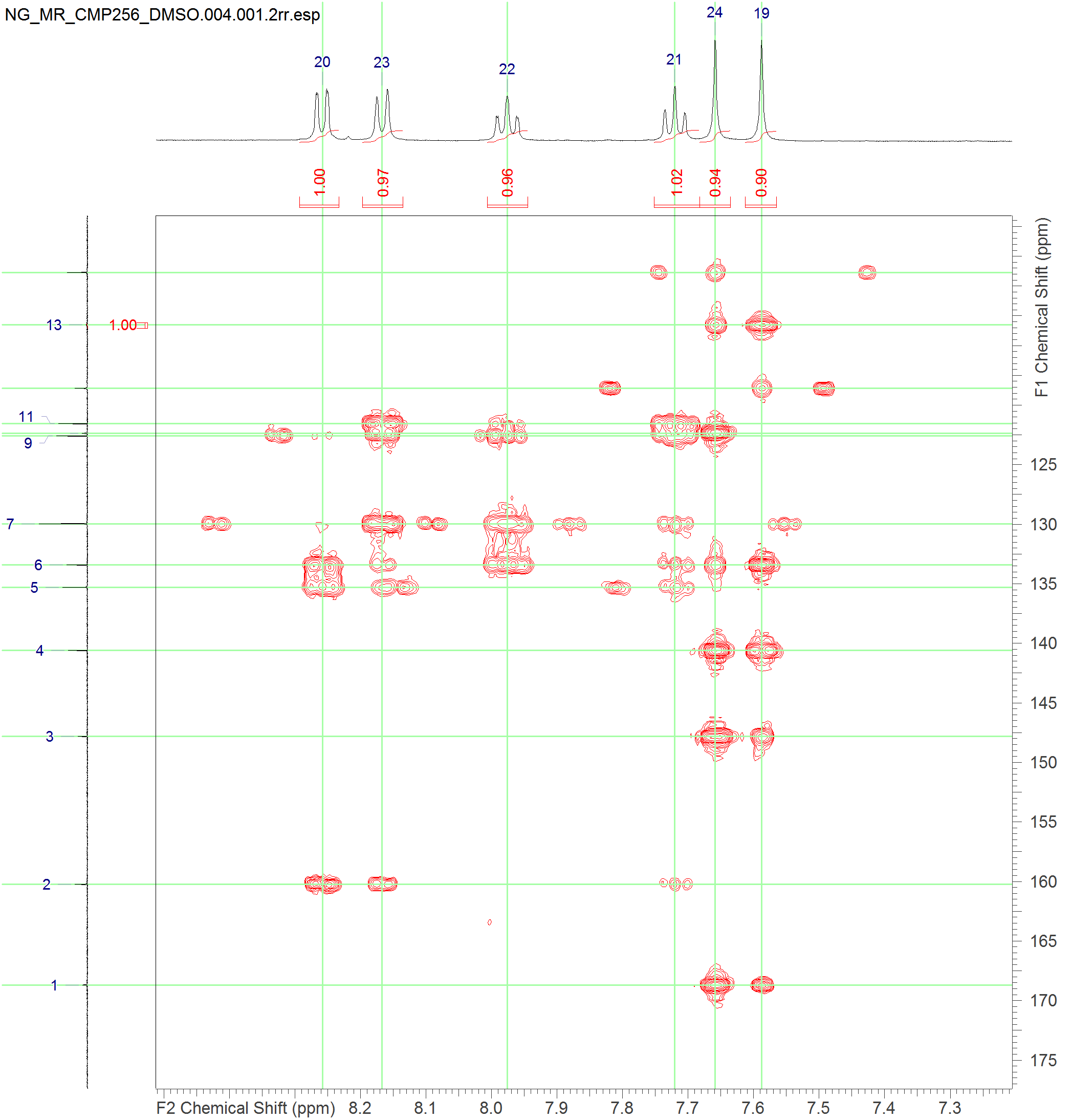


Supplementary Figure 12. HMBC spectrum (DMSO-d_6_) of benzanthric acid (7.3 ppm – 8.5 ppm & 105 ppm – 175 ppm).


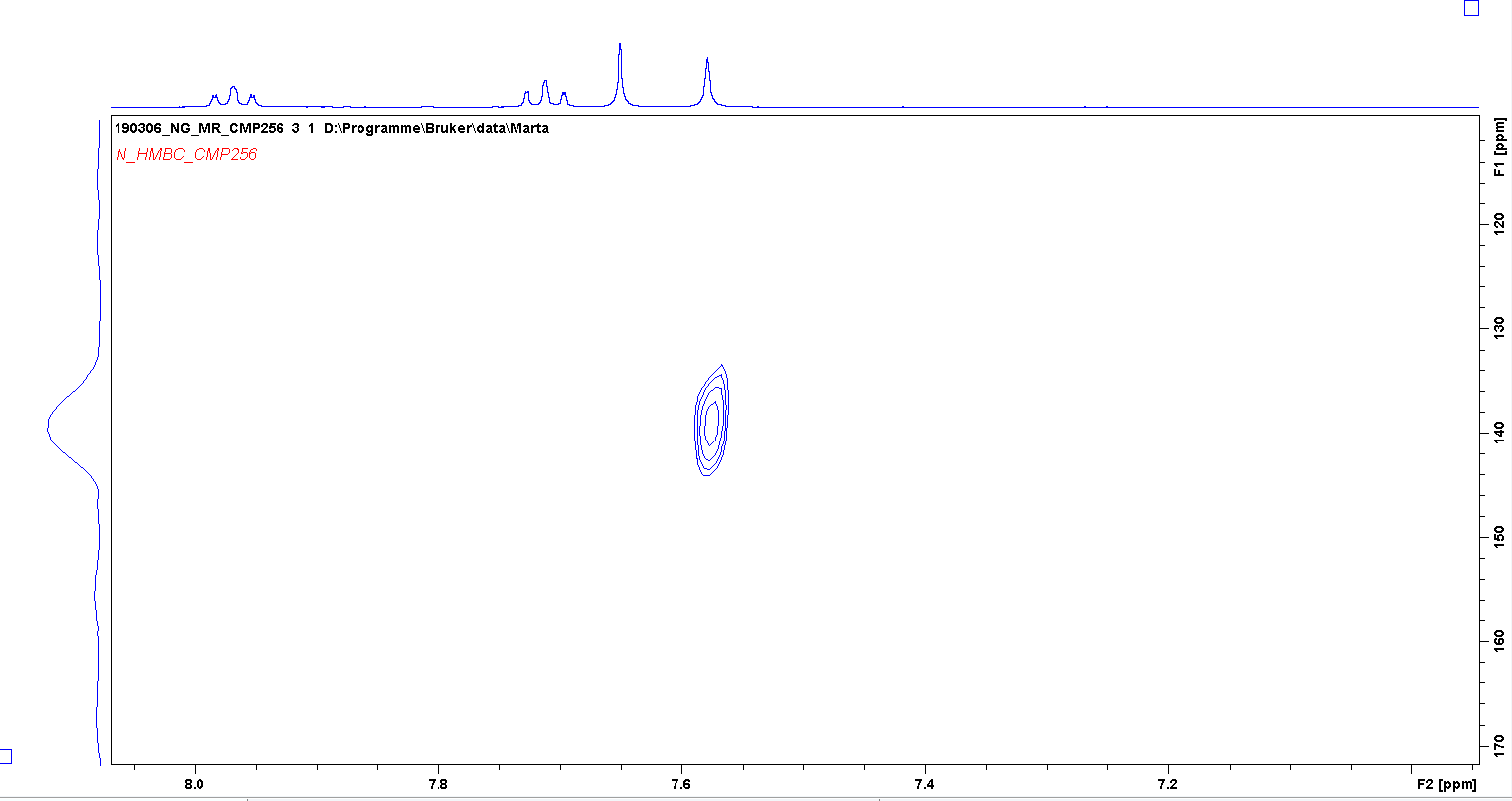


Supplementary Figure 13. ^15^N-HMBC spectrum (DMSO-d_6_) of benzanthric acid.


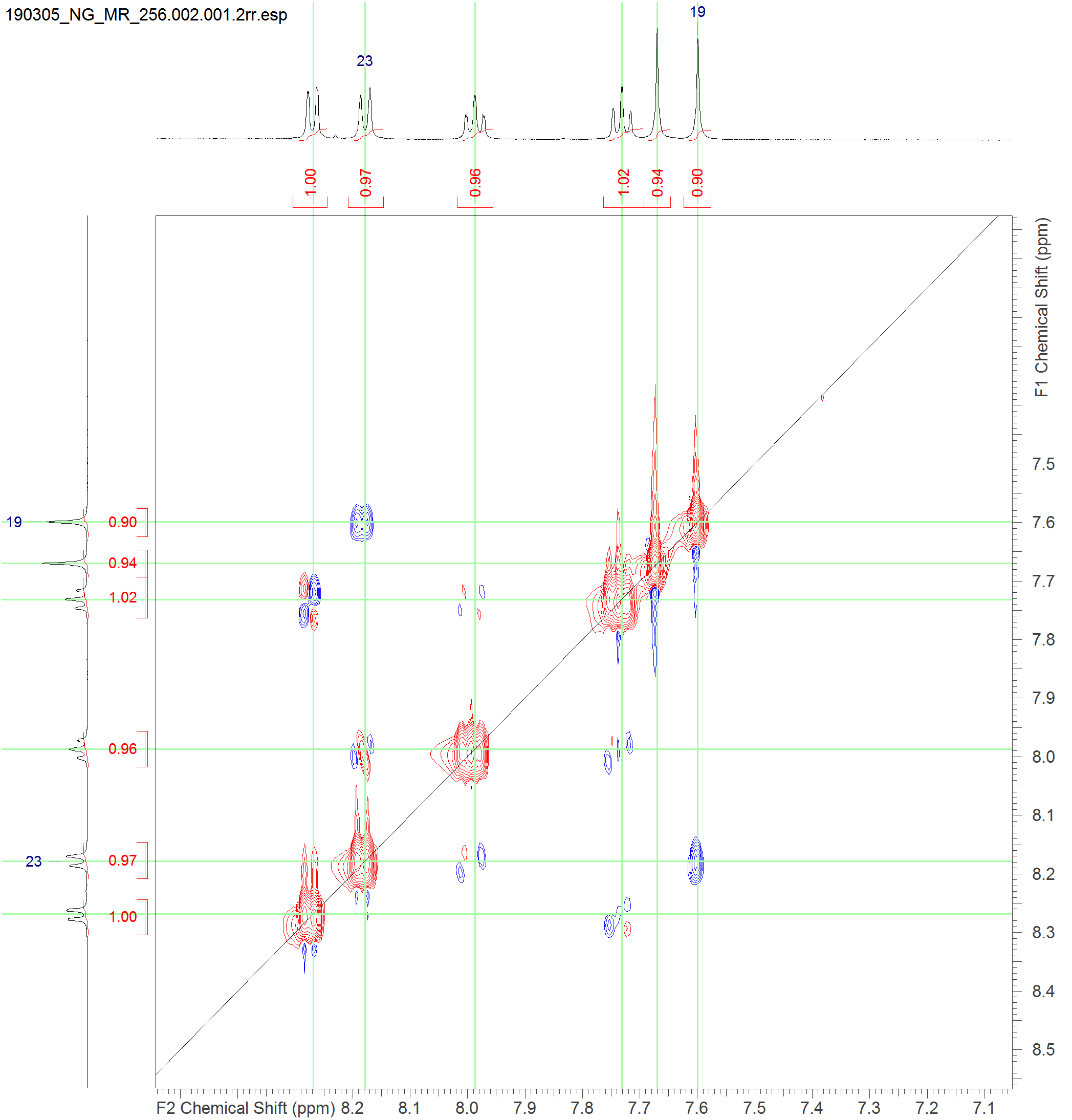


Supplementary Figure 14. ROESY spectrum (DMSO-d_6_) of benzanthric acid.


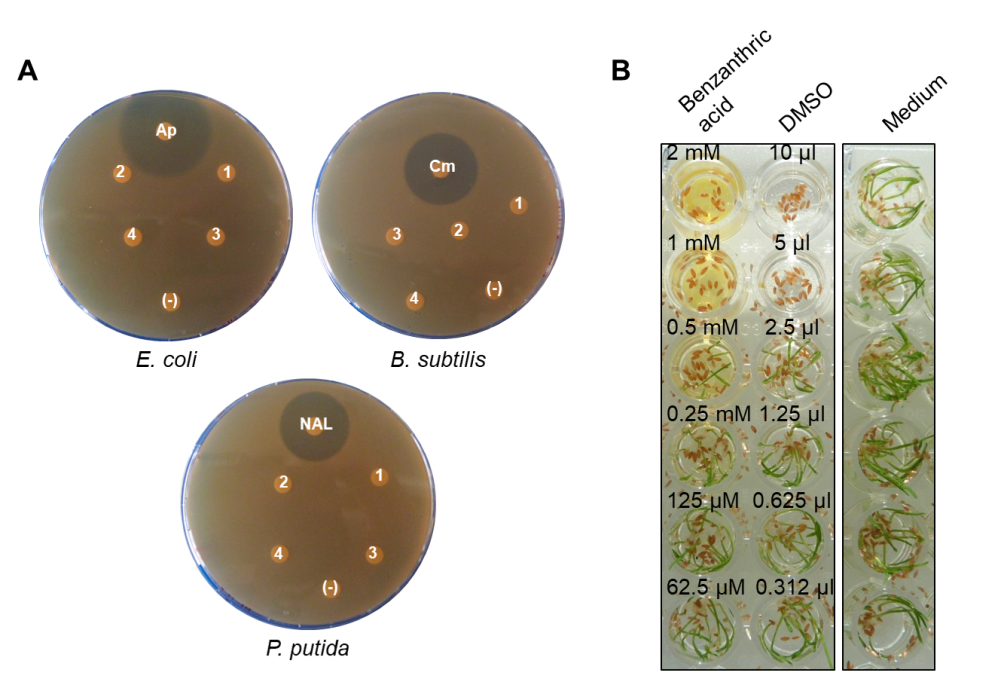


Supplementary Figure 15. Antimicrobial and herbicidal bioactivity tests. (A) Disk diffusion assay against growth of *Escherichia coli*, *Bacillus subtilis*, and *Pseudomonas putida*. Paper disks 1, 2, 3, and 4 were coated with 100 μg, 50 μg, 10 μg, and 0.5 μg, respectively, of benzanthric acid solved in DMSO. Ap: ampicillin; Cm: chloramphenicol; NAL: nalidixic acid. (-): negative control (DMSO). (B) Herbicidal test against seed germination of the monocot grass plant *Agrostis stolonifera*. Decreasing concentrations of benzanthric acid solved in DMSO were applied (first column). Identical volumes of solvent without benzanthric acid were applied (second column). Growth inhibitory effect of DMSO is observed at volumes of 10 μl and 5 μl. Minimal medium for plant growth (see section 2.7) was used as a negative control (third column).

# References

Flett, F., V. Mersinias and C. P. Smith (1997). "High efficiency intergeneric conjugal transfer of plasmid DNA from *Escherichia coli* to methyl DNA-restricting streptomycetes." FEMS Microbiology Letters **155**(2): 223-229.

Grant, S. G., J. Jessee, F. R. Bloom and D. Hanahan (1990). "Differential plasmid rescue from transgenic mouse DNAs into *Escherichia coli* methylation-restriction mutants." Proceedings of the National Academy of Sciences, USA **87**(12): 4645-4649.

Hahn, D. R., P. R. Graupner, E. Chapin, J. Gray, D. Heim, J. R. Gilbert, et al. (2009). "Albucidin: a novel bleaching herbicide from *Streptomyces albus* subsp. *chlorinus* NRRL B-24108." Journal of Antibiotics **62**(4): 191-194.

Myronovskyi, M., B. Rosenkranzer, S. Nadmid, P. Pujic, P. Normand and A. Luzhetskyy (2018). "Generation of a cluster-free *Streptomyces albus* chassis strains for improved heterologous expression of secondary metabolite clusters." Metabolic Engineering **49**: 316-324.

Rodriguez Estevez, M., M. Myronovskyi, N. Gummerlich, S. Nadmid and A. Luzhetskyy (2018). "Heterologous expression of the nybomycin gene cluster from the marine strain *Streptomyces albus* subsp. *chlorinus* NRRL B-24108." Marine Drugs **16**(11).
